# Organization of Multisynaptic Inputs to the Dorsal and Ventral Dentate Gyrus: Retrograde Trans-Synaptic Tracing with Rabies Virus Vector in the Rat

**DOI:** 10.1371/journal.pone.0078928

**Published:** 2013-11-06

**Authors:** Shinya Ohara, Sho Sato, Ken-Ichiro Tsutsui, Menno P. Witter, Toshio Iijima

**Affiliations:** 1 Division of Systems Neuroscience, Tohoku University Graduate School of Life Sciences, Sendai, Japan; 2 Kavli Institute for Systems Neuroscience and Centre for Neural Computation, Norwegian University of Science and Technology (NTNU), Trondheim, Norway; Thomas Jefferson University, United States of America

## Abstract

Behavioral, anatomical, and gene expression studies have shown functional dissociations between the dorsal and ventral hippocampus with regard to their involvement in spatial cognition, emotion, and stress. In this study we examined the difference of the multisynaptic inputs to the dorsal and ventral dentate gyrus (DG) in the rat by using retrograde trans-synaptic tracing of recombinant rabies virus vectors. Three days after the vectors were injected into the dorsal or ventral DG, monosynaptic neuronal labeling was present in the entorhinal cortex, medial septum, diagonal band, and supramammillary nucleus, each of which is known to project to the DG directly. As in previous tracing studies, topographical patterns related to the dorsal and ventral DG were seen in these regions. Five days after infection, more of the neurons in these regions were labeled and labeled neurons were also seen in cortical and subcortical regions, including the piriform and medial prefrontal cortices, the endopiriform nucleus, the claustrum, the cortical amygdala, the medial raphe nucleus, the medial habenular nucleus, the interpeduncular nucleus, and the lateral septum. As in the monosynaptically labeled regions, a topographical distribution of labeled neurons was evident in most of these disynaptically labeled regions. These data indicate that the cortical and subcortical inputs to the dorsal and ventral DG are conveyed through parallel disynaptic pathways. This second-order input difference in the dorsal and ventral DG is likely to contribute to the functional differentiation of the hippocampus along the dorsoventral axis.

## Introduction

The hippocampus, well known for its critical involvement in episodic memory and spatial navigation [1]–[4], also mediates the detection of novel or unexpected stimuli, mediates changes in hormonal regulation, and is involved in emotional responses and anxiety [5]–[7]. These various functional processes have been related to different portions along the longitudinal axis of the hippocampus. Experimental findings indicate that dorsal or septal portions contribute to the efficient processing of spatial information and that dysfunction of the dorsal portion is associated with spatial deficits. The ventral or temporal portion of the hippocampus processes information related to motivation, emotion, and homeostatic state of the animal. Dysfunction in the ventral hippocampal region is associated with affective and psychotic disorders [8]–[12].

The main connections of the hippocampus have been reported to show a topographical organization that is in line with these behavioral dorsoventral differences. The best documented are the reciprocal connections of the hippocampus with the entorhinal cortex, where it has been shown that a dorsolateral to ventromedial entorhinal origin of projections maps onto a dorsoventral termination of those projections. This longitudinal organization of these inputs is characteristic of the projections to all subfields of the hippocampus [8], [13]–[16]. The organization of the return projections from CA1 and the subiculum is consistent with this topography [17]–[19]. Other inputs and outputs show similar topographical organization [20]; a mediolateral axis of origin in the medial septum, for example, is related to a dorsoventral terminal distribution in the hippocampus [21], [22]. It has also been reported that the inputs to these two main sources of direct input to the hippocampus, i.e., the entorhinal cortex and the medial septum, are themselves topographically organized [23], [24]. Whether these organizational patterns are in any way related, however, is not known.

Viral tracers that can be transferred retrogradely across synapses, such as rabies virus or pseudorabies virus, allow this question to be addressed efficiently and with a high level of precision [25], [26]. In the present study we analyzed monosynaptic and disynaptic inputs to the dentate gyrus (DG) in the rat by using recombinant rabies virus vectors [27]–[29]. The DG is a main point of entry to the hippocampal network, and details about the chain of input connections to it are essentially unknown. In a recent study, a similar approach was used to study monosynaptic and disynaptic inputs to CA1 using the pseudorabies virus-Bartha strain [30]. That study focused on disynaptic inputs from medial prefrontal cortical regions such as the infralimbic, prelimbic, anterior cingulate, and retrosplenial cortices, which likely to be mediated through the entorhinal cortex layer III [20] or through midline and anterior domains of the thalamus [31]. In the present study we used a comparable approach but focused on inputs to the DG instead of CA1. Our study is also more elaborate in that we examined the full extent of the entorhinal cortex, including both lateral and medial subdivisions along their rostrocaudal extent, and here we report both cortical and subcortical mono- and disynaptic inputs. To compare the differences between the multisynaptic inputs to the dorsal and ventral DG precisely, we additionally used a dual trans-synaptic tracing method that can label two different neural circuits in the same experiment [28], [29]. Our data indicate that for most of the cortical and subcortical inputs to DG, parallel input streams do exist in the brain, such that cells in indirect-input areas show a clear topographical distribution related to the final targets of the input streams in the dorsal and ventral DG.

## Materials and Methods

### Surgical procedures and virus injections

A total of 19 young adult male Wistar rats weighing 200–230 g were used in this study. All experiments with injections of recombinant rabies virus vectors were carried out in a special laboratory (biosafety level 2) designated for *in vivo* infectious experiments. All experiments were approved by the Center for Laboratory Animal Research, Tohoku University, and all experiments were conducted according to the Guidelines of the National Institutes of Health and the Tohoku University Guidelines for Animal Care and Use.

Rats were deeply anaesthetized with ketamine (80.0 mg/kg, i.p.) and xylazine (0.8 mg/kg, i.p.) and were mounted in a stereotaxic frame. The skull was exposed, and a small burr hole was drilled above the injection site. The viral vectors we used as tracers were injected at different dorsoventral levels of the dentate gyrus, levels based on the atlas of Paxinos and Watson [32], by means of a glass micropipette (tip diameter  = 20–40 µm) connected to a 1 µl Hamilton microsyringe.

Nine rats received injection of either 100 nl of rHEP5.0-CVSG-mRFP [27] (7.0×10^8^ FFU/ml) or 100–200 nl of rHEP5.0-CVSG-EGFPx2 [29] (5.0×10^8^ FFU/ml) into the dorsal DG. Seven rats received injection of either 100 nl of rHEP5.0-CVSG-mRFP (7.0×10^8^ FFU/ml), 100–200 nl of rHEP5.0-CVSG-EGFPx2 (5.0×10^8^ FFU/ml), or 200 nl of rHEP5.0-CVSG-LynVenusx2 [27] (2.0×10^8^ FFU/ml) into the ventral DG. In another five rats, 100–200 nl of rHEP5.0-CVSG-mRFP (7.0×10^8^ FFU/ml) was injected into the dorsal DG and 100–200 nl of rHEP5.0-CVSG-EGFPx2 (5.0×10^8^ FFU/ml) was injected into the ventral DG. In two rats, 200 nl of rHEP5.0-CVSG-mRFP (7.0×10^8^ FFU/ml) and 200 nl of rHEP5.0-CVSG-EGFPx2 (5.0×10^8^ FFU/ml) were injected into adjacent sites (AP −3.7 and AP −5.2) of the dorsal DG. Each virus was injected along with 1% of pontamine sky blue in order to mark the injection site. After the injection, at 20 nl per minute, the pipette was left in place for another 30 minutes before it was withdrawn. After all injections were completed, the wound was sutured and the animal was monitored for recovery from anesthesia before being returned to its home cage. Throughout the survival times the rats were kept inside a small safety cabinet.

### Immunohistochemistry and Analysis

After survival periods ranging from 3 to 5 days, the animals were deeply anaesthetized with sodium pentobarbital (100 mg/kg, i.p.) and perfused transcardinally with 10% sucrose in 0.1 M PB followed by 4% paraformaldehyde in 0.1 M phosphate buffer. The brains were removed from the skulls, postfixed in 4% paraformaldehyde in 0.1 M PB for 4 hours at 4°C and then cryoprotected in PB containing 30% sucrose for at least 48 hours at 4°C. The brains were cut into 50 µm coronal sections on a freezing microtome, and sections for processing were collected in four equally spaced series.

In order to visualize the infected neurons in the single-virus injection samples, one series of sections was immunohistochemically processed for the expressed fluorescent protein (mRFP, EGFP, or Lyn-Venus). Floating sections were washed in phosphate-buffered saline (PBS), soaked with PBS containing 5% normal goat serum and 0.1% Triton-X 100 for an hour at room temperature, and then incubated overnight at 4°C with rabbit anti-RFP antibody (1∶400; Molecular Probes) or rabbit anti-GFP antibody (1∶3000; Molecular Probes) dissolved in PBS containing 5% normal goat serum and 0.1% Triton-X 100. The sections were then incubated for 2 hours at room temperature in biotinylated goat anti-rabbit IgG antibody (1∶400; Jackson ImmunoResearch) diluted in PBS containing 0.1% Triton X-100 (PBT), after which they were reacted with the avidin-biotin-peroxidase complex (ABC Elite; Vector laboratories) for another 4 hours. For visualization of the antigen, the sections were reacted in PBS containing 0.04% diaminobenzidine (DAB) and 0.002% hydrogen peroxide.

After washes in PBS the sections were mounted on gelatin-coated glass slides, air-dried, dehydrated in ethanol, cleared in xylene, and coverslipped with mounting medium (Mount Quick; Cosmo Bio). The second series of sections was Nissl-stained with thionin and used to establish cytoarchitectonic borders. Sections were examined under an Olympus Provis AX70 microscope (Olympus) and photographed using an AxioCam MRc 5 Zeiss digital camera (Carl Zeiss) and Axiovision image processing software (Carl Zeiss).

A double-labeling immunofluorescence procedure was used to visualize mRFP- and EGFP-labeled neurons in the dual-virus injection samples. After being washed in PBS, floating sections were immersed in PBS containing 5% normal goat serum and 0.1% Triton-X 100 for an hour at room temperature and then incubated overnight at 4°C with rabbit anti-mRFP IgG (1∶400; Molecular Probes) and mouse anti-GFP IgG (1∶400; Molecular Probes) dissolved in PBS containing 5% normal goat serum and 0.1% Triton-X 100. They were then washed and permeabilized in PBT and incubated for 2 hours at room temperature in Cy3-conjugated goat anti-rabbit IgG (1∶400; Jackson ImmunoResearch) and Alexa488-conjugated goat anti-mouse IgG (1∶400; Jackson ImmunoResearch) diluted in PBT. The sections were counterstained with Hoechst 33258 (1∶1000; Dojindo), coverslipped, examined under a Zeiss Axiovert 200 M microscope, and photographed using an AxioCam MRm digital camera (Carl Zeiss) and Axiovision image processing software (Carl Zeiss). To examine the dual-labeled neurons, images were captured at a fixed Z-level by using a laser scanning confocal microscope (LSM 5 Exciter, Carl Zeiss).

To compare the topographical labeling patterns of neurons innervating either the dorsal or ventral DG, the center of mass of the labeled neurons was obtained by averaging their positions. The results from different animals were superimposed onto a coronal atlas by using anatomical landmarks to normalize the positions of the labeled neurons. To examine the center of mass of labeled neurons in the piriform cortex and endopiriform cortex along the rostrocaudal axis, the positions of labeled neurons from four coronal sections (AP +2.05, AP +0.40, AP −1.25, AP −2.90) were averaged.

All numerical data are expressed as mean values ± the SEM. The statistical significance of differences between means along the dorsoventral, mediolateral or rostrocaudal axes was evaluated by using a two-tailed Student's t-test performed using Prism (Graphpad Software Inc., San Diego, CA).

## Results

**Figure 1:**
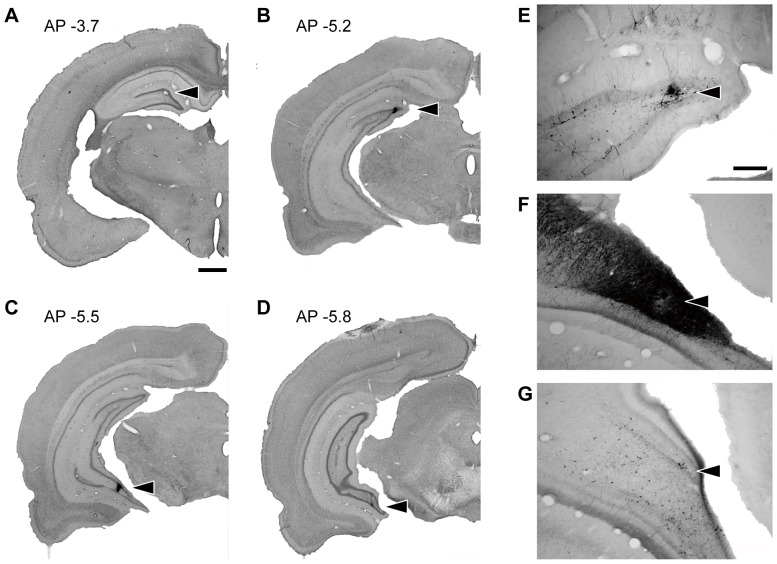
Representative injection sites in the dorsal or ventral DG. **A–D**: Nissl-stained sections with injection either in the dorsal DG at AP −3.7 (A), AP −5.2 (B), the ventral DG and CA3 region (C), or the ventral DG (D). The injection site was marked by pontamine sky blue which was co-injected with the viral vector (arrowhead). **E**–**G**: Photomicrograph of labeled neurons around the injection site. Small numbers of labeled neurons were detected in the dorsal and ventral DG injection site (E, G), whereas strong labeling was seen when the injection hit the CA3 region (F). Scale bar  = 1000 µm in A (also applies to B–D) and 250 µm in E (also applies to F, G).

To investigate multi-synaptic input to dorsal and ventral levels of the DG, we injected recombinant rabies virus vectors at different dorsoventral levels of the DG. The locations of the injections in four representative cases are shown in Figure 1. The center of the injection site was determined by the presence of the pontamine sky blue injected along with the viral vector. Since our rabies viral vector does not infect glial cells or the granule cells in the DG, labeled neurons were sparse when the injection site was in the DG, and thus the spread of virus within the DG therefore could not be determined (Fig. 1E, G). When the injection involved the CA3 region, in contrast, many strongly labeled neurons were seen there (Fig. 1F).

For the dorsal DG injections the virus was injected either at AP −3.7 (Fig. 1A) or at AP −5.2 (Fig. 1B). Although different dorsoventral levels of the DG are known to receive inputs from topologically different domains of the entorhinal cortex [33], the labeling patterns in the entorhinal cortex differed little between the two groups. Thus we put all these samples together as dorsal DG injection samples. For the ventral DG injections the virus was injected at the ventral pole of the DG (Fig. 1C, D). In some cases the pontamine sky blue labeling was seen not only in the ventral DG but also in CA3 (Fig. 1C). Since the labeling patterns in samples with injection sites in the DG and DG/CA3 were similar, all the data for those samples were taken together in this study.

### Single-virus injection

To determine the first- and second-order projection area, we first injected recombinant rabies virus vectors (rHEP5.0-CVSG-EGFPx2, rHEP5.0-CVSG-mRFP, or rHEP5.0-CVSG-LynVenusx2) into either the dorsal or the ventral DG and examined the distribution of labeled neurons at different post-inoculation survival times. With these viral vectors the optimal survival time for labeling first-order projection neurons is three days. It takes another 2 days for the vector to cross one synapse and infect the second-order projection neurons [29].

**Figure 2:**
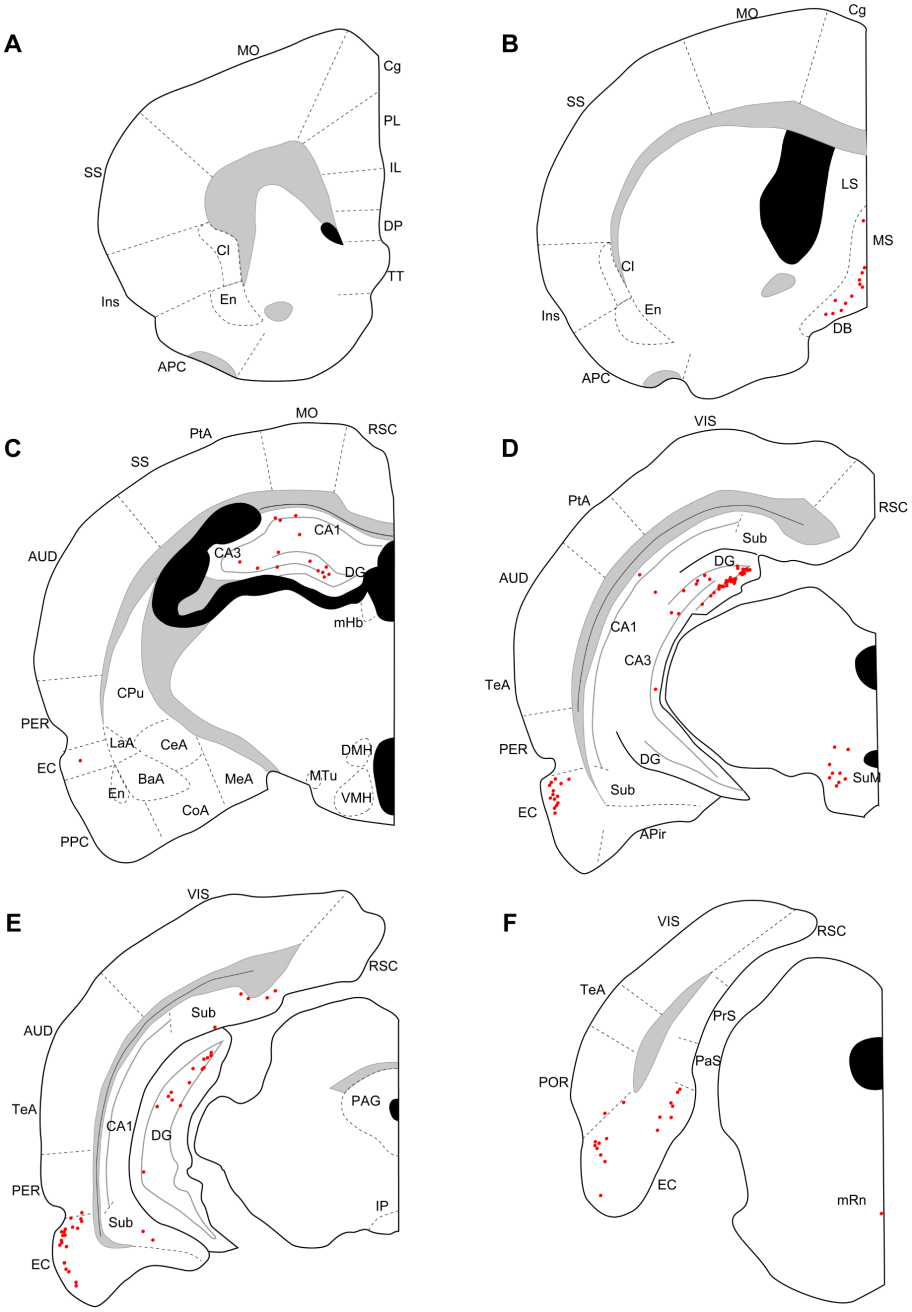
Distribution of retrogradely labeled neurons three days after virus injections into dorsal DG. Series of coronal sections (organized from rostral to caudal) for a rat surviving three days after dorsal DG injection. APC, anterior piriform cortex; APir, amygdalopiriform transition; AUD, auditory cortex; BaA, basal complex of amygdala; CeA, central amygdaloid nucleus; Cg, cingulate cortex; Cl, claustrum; CoA, cortical amygdaloid nucleus; CPu, caudate putamen; DB, diagonal band; DMH, dorsomedial hypothalamic nucleus; DP, dorsal peduncular cortex; En, endopiriform nucleus; EC, entorhinal cortex; IL, infralimbic cortex; Ins, insular cortex; IP, interpedunclular nucleus; LaA, lateral amygdaloid nucleus; LS, lateral septum; MeA, medial amygdaloid nucleus; mHb, medial habenular nucleus; mRn, median raphe nucleus; MO, motor cortex; MS, medial septum; MTu, medial tuberal nucleus; PAG, periaqueductal gray; PaS, parasubiculum; PER, perirhinal cortex; PL, prelimbic cortex; POR, postrhinal cortex; PPC, posterior piriform cortex; PrS, presubiculum; PtA, parietal association cortex; RSC, retrosplenial cortex; SS, somatosensory cortex; Sub, subiculum; SuM, supramammillary nucleus; TeA, temporal association cortex; TT, tenia tecta; VIS, visual cortex; VMH, ventromedial hypothalamic nucleus.

**Figure 3:**
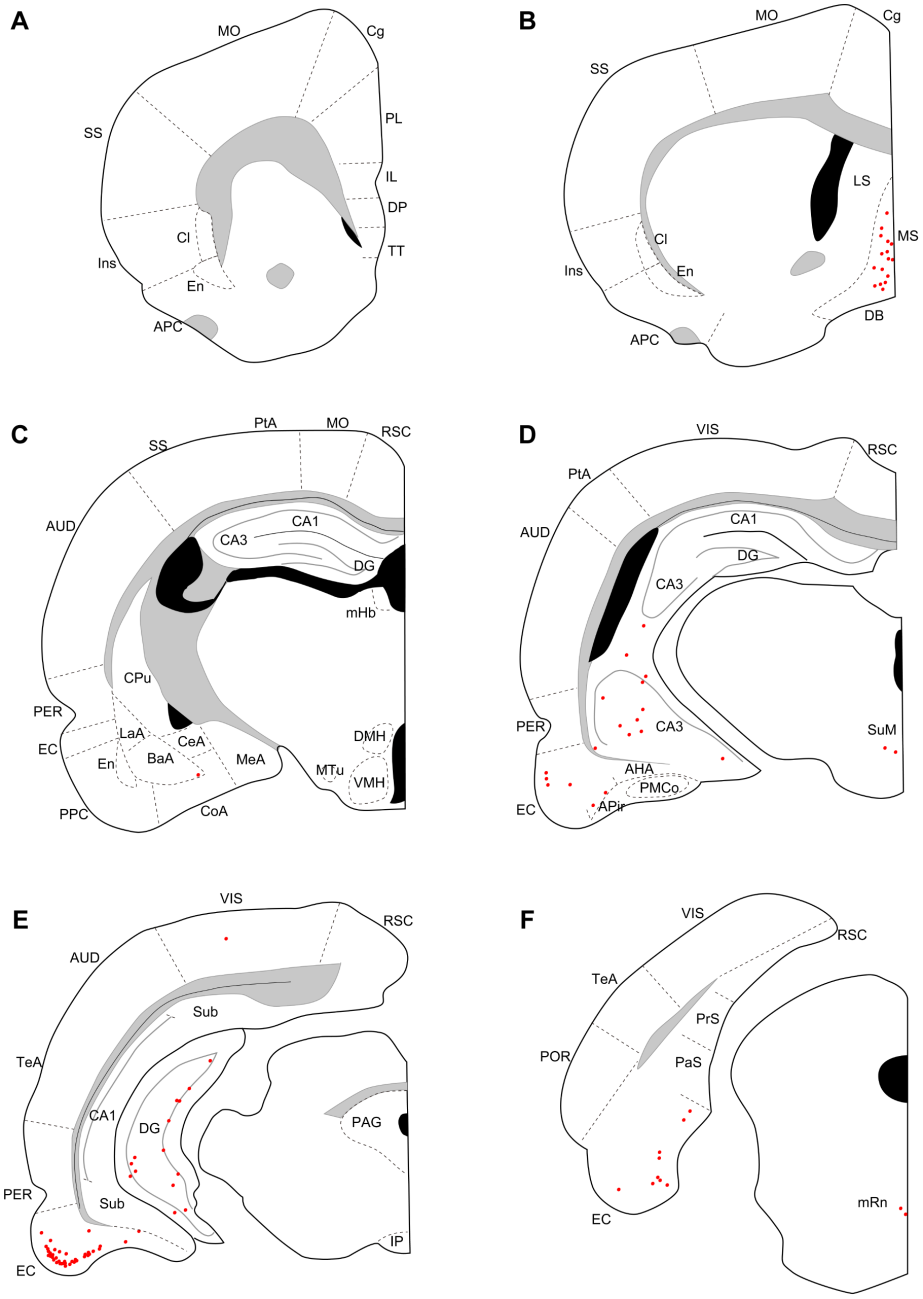
Distribution of retrogradely labeled neurons three days after virus injections into ventral DG. Series of coronal sections (organized from rostral to caudal) for a rat surviving three days after ventral DG injection. AHA, amygdalohippocampal area; PMCo, posteromedial cortical nucleus of the amygdala. Remainder of abbreviations the same as in Figure 2.

**Figure 4:**
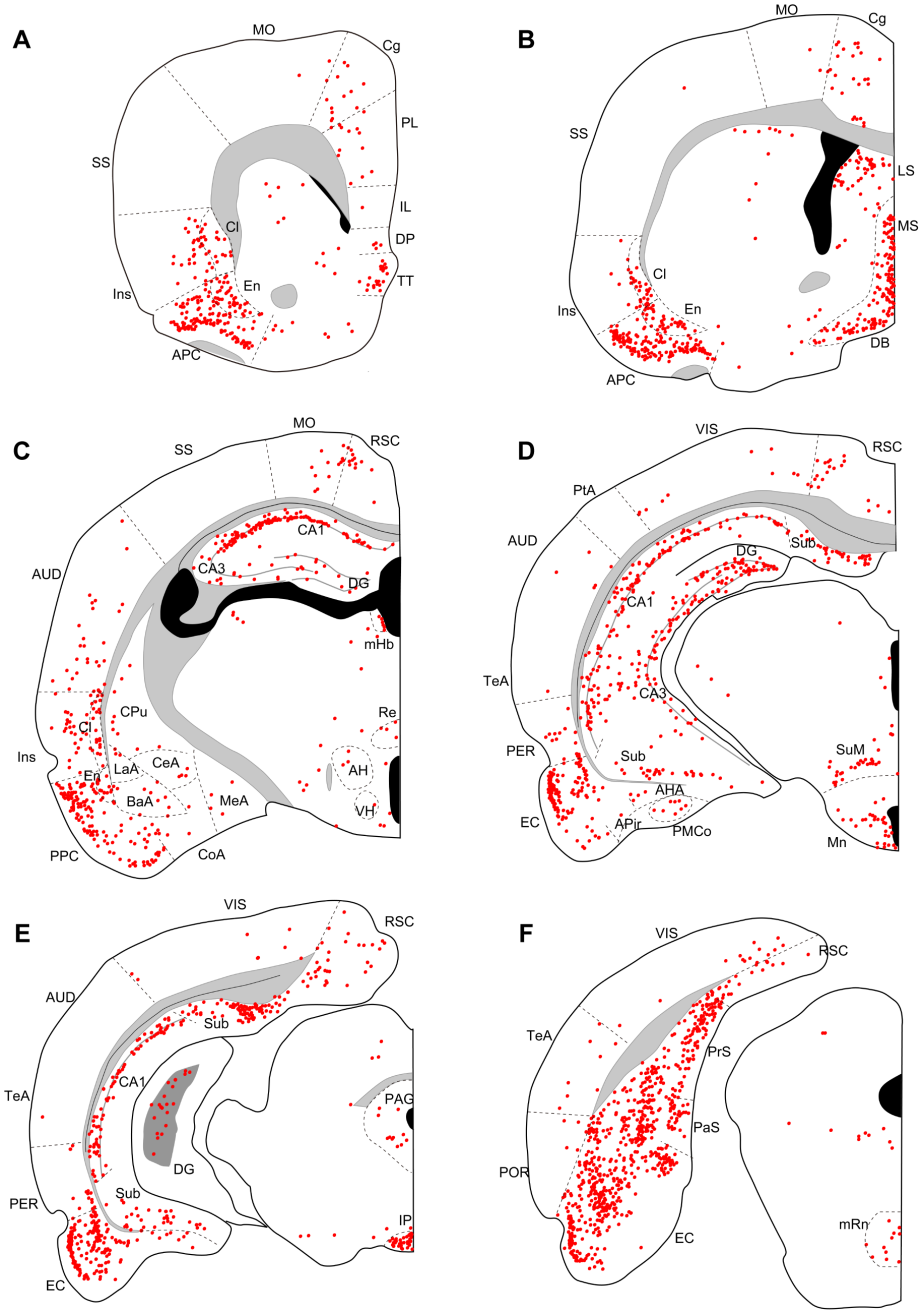
Distribution of retrogradely labeled neurons five days after virus injections into dorsal DG. Series of coronal sections (organized from rostral to caudal) for a rat surviving five days after dorsal DG injection. AH, anterior hypothalamic area; Mn, mammillary nucleus; Re, reuniens thalamic nucleus; VH, ventral hypothalamic nucleus. Remainder of abbreviations the same as in Figure 2 and 3.

**Figure 5:**
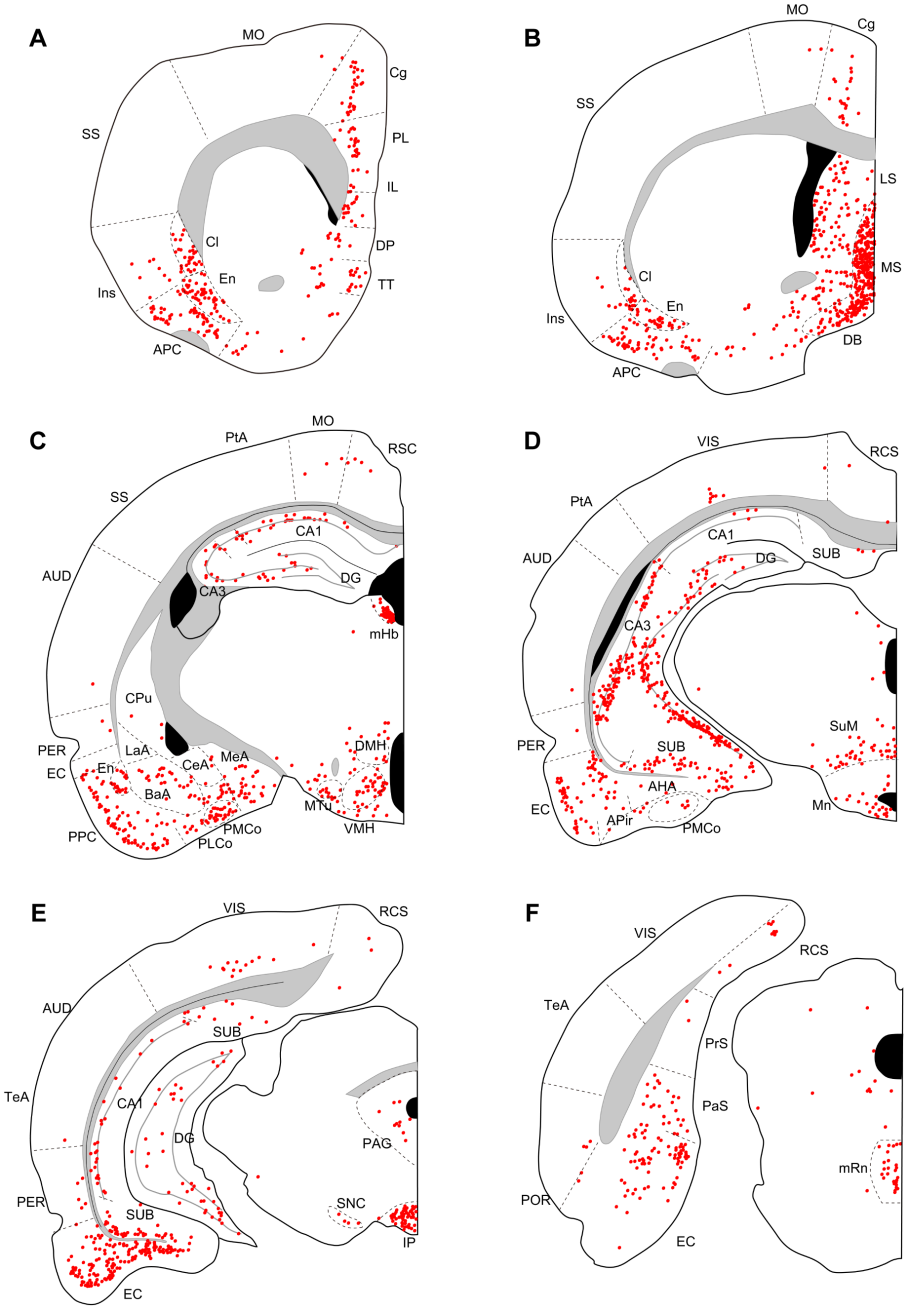
Distribution of retrogradely labeled neurons five days after virus injections into ventral DG. Series of coronal sections (organized from rostral to caudal) for a rat surviving five days after ventral DG injection. For abbreviations, see list. PLCo, posterolateral cortical nucleus of the amygdala; SNC, substantia nigra pars compacta. Remainder of abbreviations the same as in Figure 2–4.

Three days after an injection into the dorsal (N = 4) or ventral DG (N = 3), many labeled neurons were seen in CA3, the entorhinal cortex, the medial septum, the diagonal band, and the supramammillary nucleus (Fig. 2, 3). In three out of four cases with dorsal DG injection, a few labeled neurons were also seen in the median raphe nucleus (Fig. 2F), interpeduncular nucleus, and claustrum. In ventral injection samples, a few labeled neurons were seen in the median raphe nucleus (Fig. 3F) in all three cases and in the interpeduncular nucleus and claustrum in one case. At five days of post-inoculation survival after a dorsal (N = 5) or a ventral DG injection (N = 4), robust labeling was also seen in other cortical and subcortical regions, such as the medial prefrontal cortex, piriform cortex, endopiriform nucleus, claustrum, lateral septum, medial habenular nucleus, interpeduncular nucleus, and median raphe nucleus (Fig. 4, 5). Since retrograde labeling in these areas was not present at three days, this pattern of labeling is thought to result from trans-synaptic labeling and thus indicate second-order projection neurons. In all samples, labeling in the perirhinal and postrhinal cortices was sparse (Fig. 4D–F, 5D–F). In three of the five cases with dorsal DG injection, dense labeling was seen in the presubiculum (Fig. 4F), where labeling was not present in samples with three-day survival (Fig. 2F). In samples with ventral DG injection but not in samples with dorsal DG injection, robust labeling was seen in the posterior cortical nucleus of the amygdala (Fig. 5C). For all first-order and second-order brain structures containing labeled cells, we saw a clear difference in the position of the labeled cells within the cortex or nucleus depending on whether the injection involved the dorsal or ventral DG. This will be described in detail in the next section.

### Dual-virus injection

**Figure 6:**
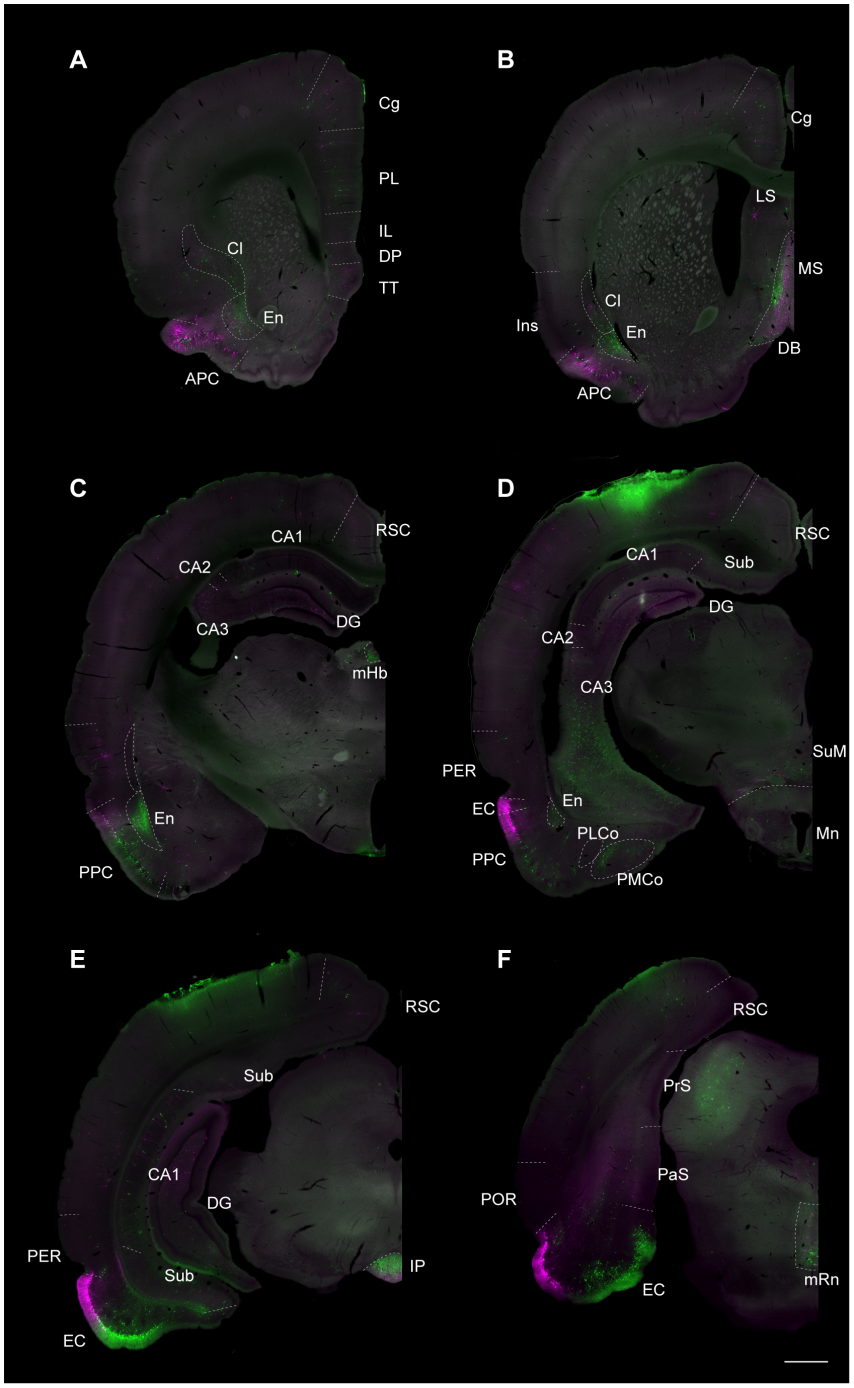
Distribution of retrogradely labeled neurons after dual-virus injections. Low-power fluorescence micrographs showing the distribution of retrogradely labeled neurons after five days survival, resulting from injections of rHEP5.0-CVSG-mRFP into the dorsal DG and rHEP5.0-CVSG-EGFPx2 into the ventral DG. **A–F**: Six representative coronal sections arranged from rostral to caudal. RFP-labeled cells are shown in magenta, and GFP-labeled cells are shown in green. Abbreviations the same as in Figure 2–5. Scale bar  = 1000 µm.

To compare the topographical organization of mono- and disynaptic inputs to the dorsal and ventral DG, we injected two recombinant rabies virus vectors expressing different fluorescent proteins into the brains of two animals. The mRFP expressing virus (rHEP5.0-CVSG-mRFP) was injected into the dorsal DG, and the EGFP expressing virus (rHEP5.0-CVSG-EGFPx2) was injected into the ventral DG. After a survival time of five days, the distribution of labeled neurons was similar to what was seen in case of single injections in either the dorsal or ventral DG (Fig. 6). Similar labeling patterns were also seen in animals with longer survival time (6 days, N = 1; 7 days, N = 2; data not shown). In our analysis of these patterns we differentiated between first- and second-order labeled neurons by using information inferred from the different survival times in the experiments with single-virus injections.

### First-order labeled neurons

**Figure 7:**
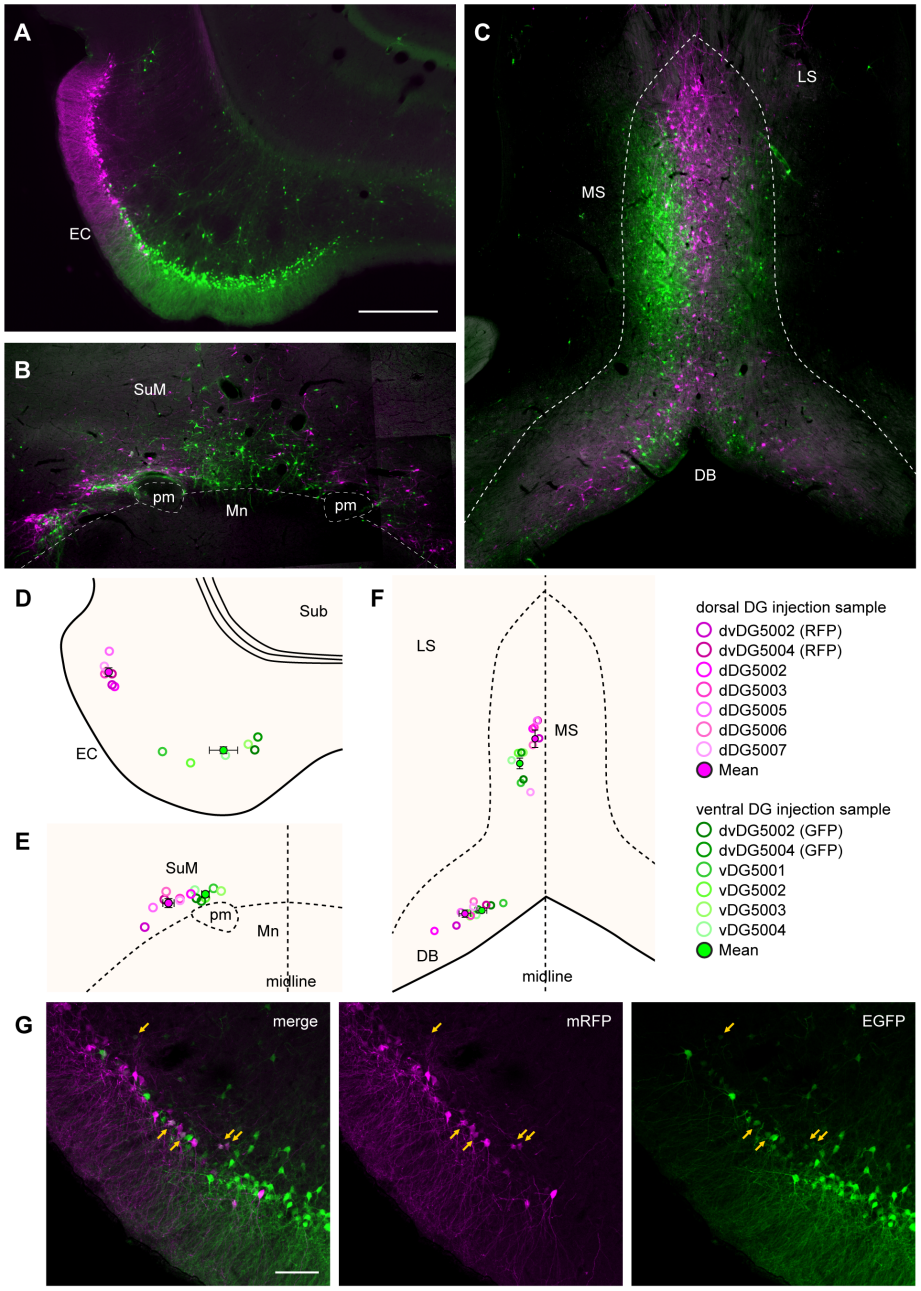
Distribution of retrogradely labeled neurons in the first-order projection area after dual-virus infection. **A**–**C**: Fluorescence micrograph of labeling in the entorhinal cortex (A), the supramammillary nucleus (B), and in the medial septum and diagonal band (C). These regions are known to have direct projection to the DG. Neurons infected by the viral vector injected to dorsal DG (RFP-labeled cells) are shown in magenta, while neurons infected by the ventral-DG-injected vector (GFP-labeled cells) are shown in green. **D**–**F**: The center of mass of labeled neurons in the entorhinal cortex (D), the supramammillary nucleus (E), and in the medial septum and diagonal band (F). The open circles indicate the centers of mass for individual samples. The filled circles and the error bar indicates the mean ± SEM across samples (N = 7 for dorsal-DG injection, and N = 6 for ventral-DG injection). The results of the dorsal-DG injection are shown in magenta while the results of the ventral-DG injection are shown in green. **G**: Fluorescence micrographs showing the labeled neurons at the border of GFP- and RFP- labeled neurons in the entorhinal cortex. Arrows indicate double-labeled neurons. pm, principle mammillary tract. Remainder of abbreviations the same as in Figure 2 and 4. Scale bar  = 500 µm in A (also applies to B, C) and 100 µm in G.

Figure 7 illustrates in detail the labeling in the first-order projection areas selected on the basis of the single-virus injection cases with a survival of three days. The viral vector injected into the dorsal DG (rHEP5.0-CVSG-mRFP) resulted in RFP-positive neurons positioned laterally in the lateral entorhinal cortex as well as laterally and caudally in the medial entorhinal cortex. In contrast, the ventrally injected viral vector (rHEP5.0-CVSG-EGFPx2) resulted in GFP-labeled neurons preferentially in medial portions of both the lateral entorhinal cortex and the medial entorhinal cortex (Fig. 6E–F, Fig. 7A). This pattern is similar to that seen following injections of a single viral vector into either the dorsal or ventral DG (Fig. 2, 4), indicating that two injections of a viral vector into a single brain structure do not seem to change the uptake and transport characteristics of either of the two vectors. In the supramammillary nucleus, GFP-labeled neurons were located more medially than RFP-labeled neurons (Fig. 7B). In the medial septum, medially located neurons were labeled with RFP and laterally positioned neurons were labeled with GFP. In the diagonal band this differential pattern of labeling is apparently reversed such that the ventrally projecting neurons are in a more ventromedial position than those that project dorsally (Fig. 6B, Fig. 7C).

To statistically evaluate whether these topographical labeling patterns are consistent in the single- and dual-virus injection samples, we performed a center of mass analysis and determined the center of the inputs to the dorsal and ventral DG (Fig. 7D– F). Overall, neurons innervating the dorsal DG were, compared with neurons innervating the ventral DG, located significantly more laterally in the entorhinal cortex (p<0.0001 by unpaired t test) and in the supramammillary nucleus (p<0.001) and significantly more medially in the medial septum (p<0.0001). In the diagonal band, although the dorsal-DG innervating neurons tended to be located more laterally than the ventral-DG innervating neurons, the difference between the two groups was not significant (p = 0.067). In these first-order projection areas, the GFP-labeled neurons and RFP-labeled neurons distributed separately and the two labels intermingled only at the border of the labeled populations. Double-labeled neurons were sparsely observed only at this narrow border area of overlapping labeled projection neurons (Fig. 5G).

### Second-order labeled neurons

**Figure 8:**
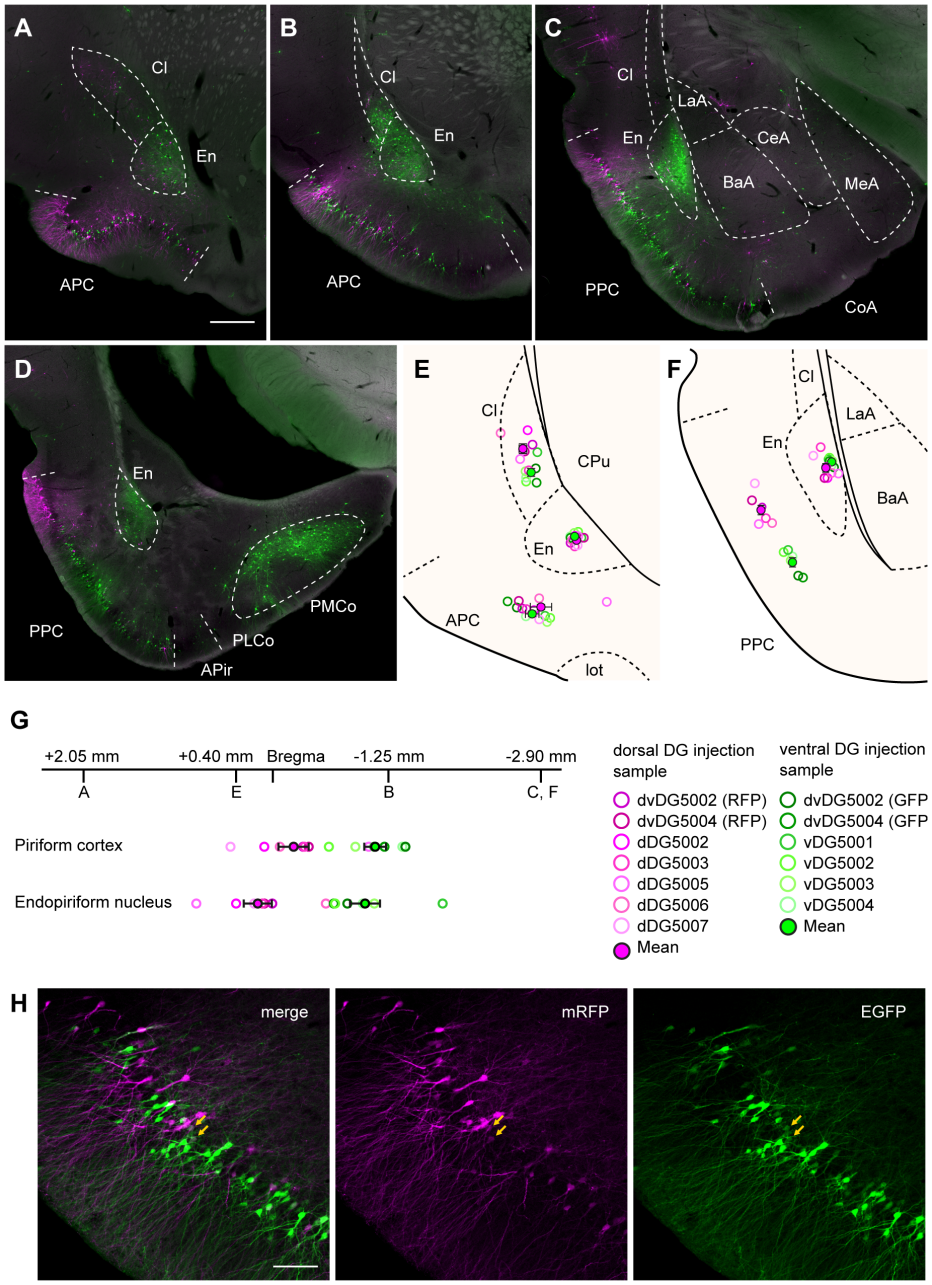
Distribution of retrogradely labeled neurons in the piriform and amygdaloid areas after dual-virus infection. **A**–**D**: Labeled neurons in coronal sections through the ventral portion of the hemisphere arranged from rostral to caudal (A, B, C, and D respectively show the labeling at AP +2.05, AP −1.25, AP −2.90 and AP −4.00). Labeled neurons were seen in the anterior piriform cortex (APC), the posterior piriform cortex (PPC), the claustrum (Cl), the endopiriform nucleus (En) and the posteromedial cortical nucleus of the amygdala (PMCo). RFP-labeled cells are shown in magenta, and GFP-labeled cells are shown in green. **E**–**F**: The center of mass of labeled neurons in a coronal section at AP +0.40 (E), and at AP −2.90 (F). The open circles indicate the centers of mass for individual samples. The filled circles and the error bar indicates the mean ± SEM across samples (N = 7 for dorsal-DG injection, and N = 6 for ventral-DG injection). The results of the dorsal-DG injection are shown in magenta while the results of the ventral-DG injection are shown in green. **G**: The center of mass of labeled neurons in the piriform cortex and endopiriform cortex along the rostrocaudal axis. The center of mass was obtained by averaging the positions of labeled neurons in four coronal sections from different position along the rostrocaudal axis (AP +2.05, AP +0.40, AP −1.25, AP −2.90). **H**: Fluorescence micrographs showing the labeled neurons in the anterior piriform cortex. Arrows indicate double-labeled neurons. lot, lateral olfactory tract. Remainder of abbreviations the same as in Figure 2, 3 and 5. Scale bar  = 500 µm in A (also applies to B–D) and 100 µm in H.

Second-order labeling was prominent in a number of cortical and subcortical brain areas. The distribution patterns of the two differently labeled populations of neurons in case of all second-order inputs showed striking conservation of the topographical organization described for the first-order inputs. This was similar to what was noted in case of the single-virus injections into the DG. In the anterior part of the brain, many labeled neurons were seen in the anterior piriform cortex, the posterior piriform cortex, the endopiriform nucleus, the claustrum, and in the posterior cortical nucleus of amygdala, all thought to be second-order input areas of the DG (Fig. 6A–D, Fig. 8). In the piriform cortex, the two populations of labeled cells showed considerable overlap but exhibited a topographical distribution (Fig. 8). More RFP-labeled neurons were observed in the anterior piriform cortex than in the posterior piriform cortex. In contrast, the number of GFP-labeled neurons increased at more caudal levels of the piriform cortex (Fig. 8A–D). As a result, the center of mass of neurons innervating the dorsal DG was significantly rostral to that of the neurons innervating the ventral DG (−0.24±0.16 mm from Bregma for dorsal injections; N = 7, −1.11±0.12 mm from Bregma for ventral injections; N = 6, p<0.01, Fig. 8G). In the posterior piriform cortex, clear topographical labeling patterns were observed along the dorsolateral-to-ventromedial axis (Fig. 8C). Neurons innervating the dorsal DG were located dorsolateral to the neurons innervating the ventral DG (p<0.0001, Fig. 8F). In the endopiriform nucleus, a comparable topographical labeling pattern was observed along the rostrocaudal axis. The majority of RFP-labeled neurons was seen rostrally in the nucleus whereas the number of GFP-labeled neurons increased at more caudal levels (Fig. 8A–D). As a result, the center of mass of the dorsal DG innervating neurons were significantly rostral to that of the ventral DG innervating neurons in the endopiriform nucleus (+0.15±0.15 mm from Bregma for dorsal injections; N = 7, −1.01±0.40 mm from Bregma for ventral injections; N = 6, p<0.001, Fig. 8G). Labeling in the claustrum was also topographically distributed such that the GFP-labeled neurons were seen ventrally and RFP-labeled neurons were mainly seen dorsally (Fig. 8A). This labeling pattern in the claustrum was significant especially in the rostral sections (p<0.001 in AP +2.05, p<0.01 in AP +0.40, p = 0.06 in AP −1.25, Fig 8E). In view of the overlap between the populations of GFP and RFP expressing neurons, we checked for the presence of double labeled neurons. Throughout the rostrocaudal axis they were sparsely present in the piriform cortex (Fig. 8H), endopiriform nucleus and the claustrum. Many GFP-labeled neurons were also seen in the posterior cortical nucleus of the amygdala, while RFP-labeled neurons were not found in this region (Fig. 8D). In the remainder of the amygdala, only very sparse labeling was seen (Fig. 8C).

**Figure 9:**
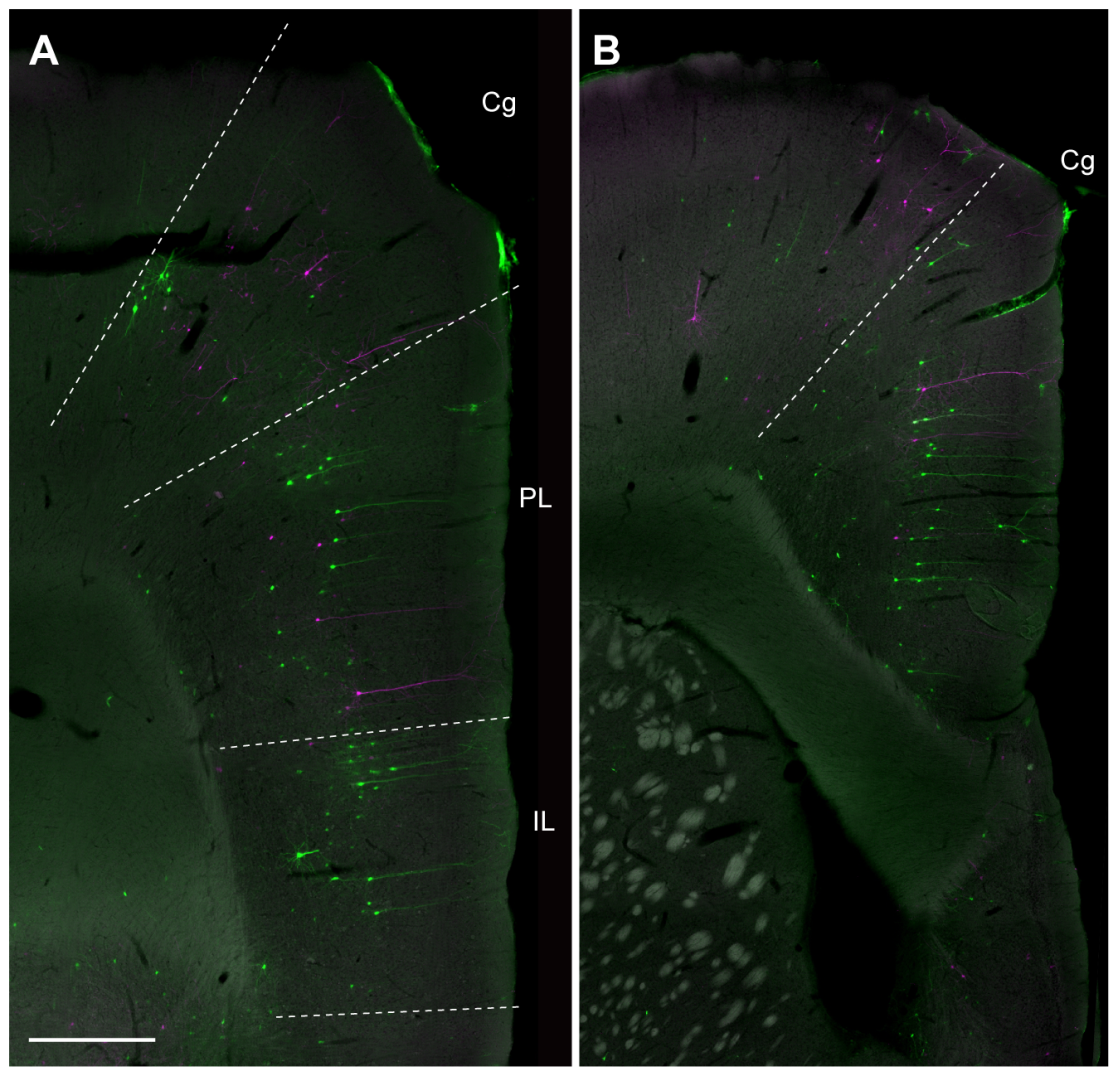
Fluorescence micrographs of retrogradely labeled neurons in the medial prefrontal cortex. **A**: Rostral section showing labeling in the prelimbic (PL), the infralimbic cortex (IL) and in the rostral part of the cingulate cortex (Cg). **B**: More caudal section showing labeling in Cg. RFP-labeled cells are shown in magenta, and GFP-labeled cells are shown in green. Scale bar  = 500 µm in A (also applies to B).

At these anterior levels we further saw retrogradely labeled neurons of both colors in the medial prefrontal cortex (Fig. 9). In the medial prefrontal prelimbic and infralimbic cortices, more GFP-labeled neurons were seen than RFP-labeled neurons. In the cingulate cortex, in contrast, both types were equally present. In these animals with two injections, labeled neurons of either color were sparse or absent in the retrosplenial cortex, similar to what was seen in case of the single injections. Double-labeled neurons were not seen in the medial prefrontal cortex.

**Figure 10:**
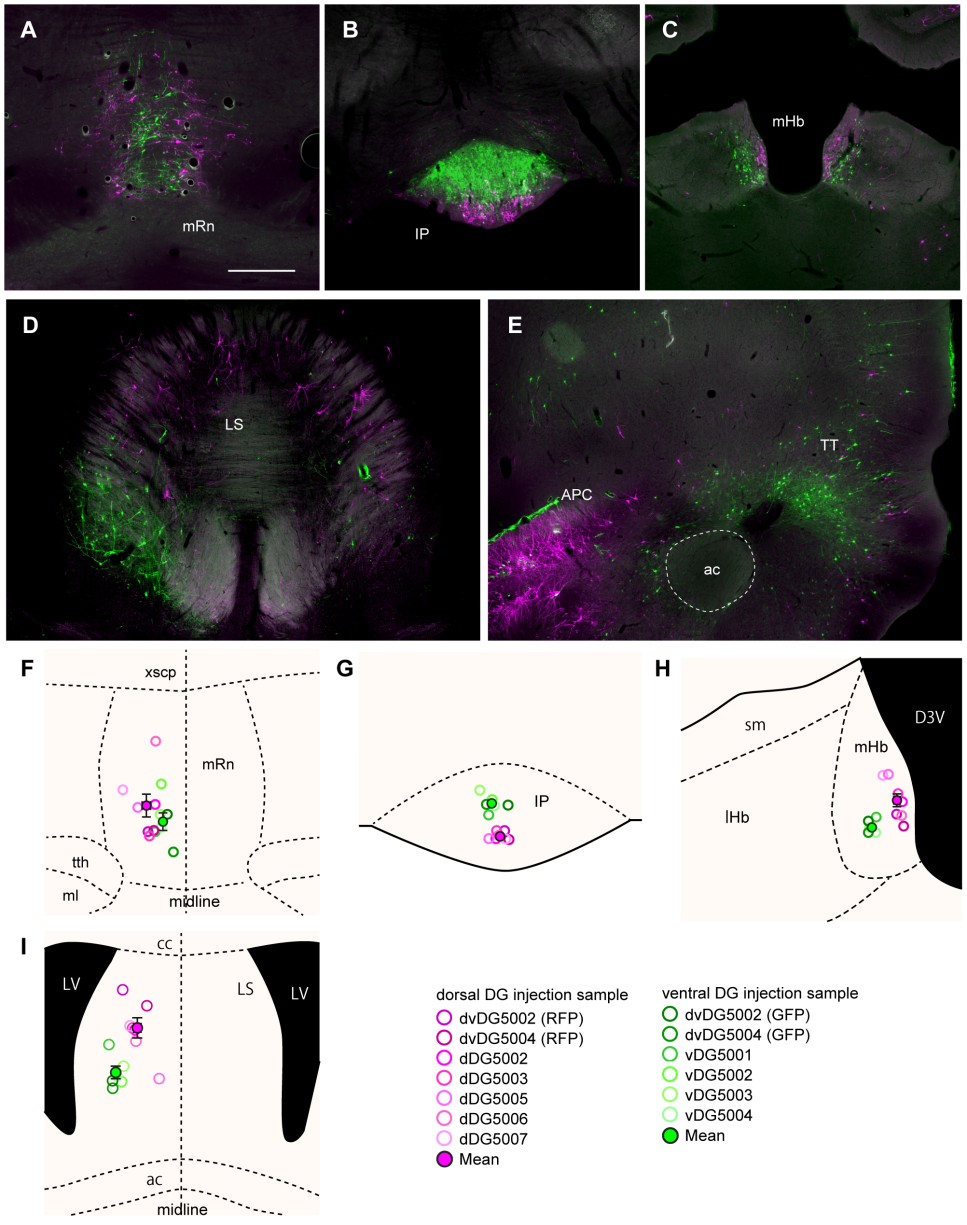
Distribution of retrogradely labeled neurons in second-order projection areas after dual-virus infection. **A**–**E**: Fluorescence micrograph of labeling in the median raphe nucleus (A), the interpeduncular nucleus (B), the medial habenula (C), the lateral septum (D), and the tenia tecta (E). RFP-labeled cells are shown in magenta, and GFP-labeled cells are shown in green. **F**–**I**: The center of mass of labeled neurons in the median raphe nucleus (F), the interpeduncular nucleus (G), the medial habenula (H), the lateral septum (I). The magenta and green open circles indicate the centers of mass from individual samples with virus injection into the dorsal- and ventral-DG respectively. The filled circles and the error bar indicates the mean ± SEM across samples (N = 7 for dorsal-DG injection, and N = 6 for ventral-DG injection). ac, anterior commissure; cc, corpus callosum; D3V, dorsal 3^rd^ ventricle; lHb, lateral habenular nucleus; LV, lateral ventricle; ml, medial lemniscus; sm, stria medullaris thalami; tth, trigeminothalamic tract; xscp, decussation of the superior cerebellar peduncle. Remainder of abbreviations the same as in Figure 2. Scale bar  = 500 µm in A (also applies to B–E).

Finally, large numbers of labeled neurons were seen in the medial raphe nucleus, the interpeduncular nucleus, the medial habenular nucleus, and in the lateral septum. In the median raphe nucleus, neurons in medial positions were labeled by GFP, while more laterally positioned neurons were labeled by RFP (Fig. 10A). In the interpeduncular nucleus, the GFP-labeled neurons were mainly seen dorsally, while RFP-labeled ones were confined ventrally (Fig. 10B). In the medial habenular nucleus, RFP-labeled neurons were located medial to the GFP-labeled neurons (Fig. 10C). In the lateral septum, again a clear topology of labeled neurons was seen in the caudal half, where GFP-labeled neurons were seen ventrally, while the RFP-labeled neurons accumulated dorsally (Fig. 10D). Finally, in the tenia tecta, GFP-positive neurons were most prominent (Fig. 10E). In the four regions that showed marked topographically distributed labeling, significant differences in the center of mass were observed between the dorsal and ventral injection groups (p<0.05 in the median raphe nucleus along the mediolateral axis, p<0.0001 in the interpeduncular nucleus along the dorsoventral axis, p<0.0001 in the medial habenular nucleus in mediolateral axis, p<0.01 in the lateral septum along the dorsoventral and mediolateral axis; Fig. 10F–I).

**Figure 11:**
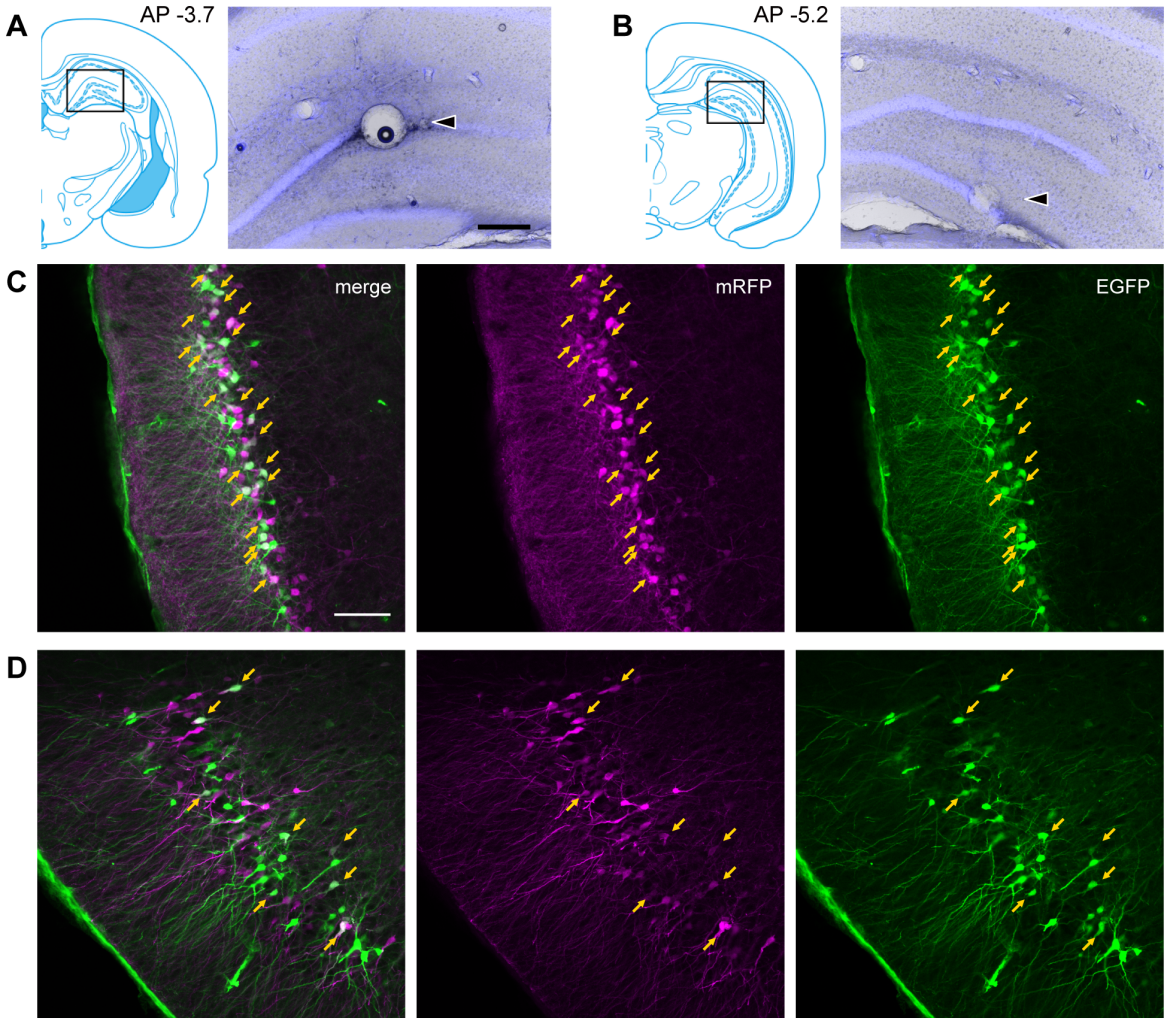
Fluorescence micrographs of retrogradely labeled neurons after injection of two viruses in the dorsal DG. **A**–**B**: The coronal atlas and the photomicrograph of the injection site. rHEP5.0-CVSG-mRFP was injected into the dorsal DG at AP −3.7 while rHEP5.0-CVSG-EGFPx2 was injected into the dorsal DG at AP −5.2. Arrowhead shows the center of the injection site. **C**–**D**: Fluorescence micrographs showing the labeled neurons in the entorhinal cortex (C) and in the anterior piriform cortex (D) after five days of survival. RFP-labeled cells are shown in magenta, and GFP-labeled cells are shown in green. Arrows indicate double-labeled neurons. Scale bar  = 300 µm in A (also applies to B) and100 µm in C (also applies to D).

Similar to what was seen in the first-order projection areas, only few double-labeled neurons were seen at the border of the two populations of GFP- and RFP-labeled neurons in these brainstem areas. The relatively sparse presence or even absence of double-labeled cells following injections of the two rabies vectors in the dorsal and ventral hippocampus is taken to be caused by a topographical organization of both first- and second-order inputs. However, the lack of double-labeled neurons may result from competition between the two vectors [29]. To control for this potential bias, we looked at double labeling when injecting the two vectors closer together in the dorsal DG. Injections of the two vectors 1.5 mm apart in the dorsal DG resulted in clearly mixed populations of first-order labeling for example in the lateral entorhinal cortex and second-order labeling in the anterior piriform cortex. Large numbers of double-labeled cells were seen in both areas (Fig. 11).

## Discussion

In this study we not only identified brain areas that provide second-order inputs to the dentate gyrus (DG) but, more important, found that these disynaptic inputs to dorsal and ventral levels of the DG showed a topographical organization maintaining that of the monosynaptic inputs to the DG [15]. We injected recombinant rabies virus vectors into either the dorsal or ventral pole of DG and allowed the animals to survive for either three or five days. We have previously shown that with the then-used rabies vectors a survival time of 3.5 to 4 days almost exclusively labeled monosynaptic inputs to the injection site while a 6-day survival time was long enough for labeling most if not all of the second-order inputs [29]. In this study we used a shorter survival time than we did in the previous study because the virus titer we used was more than 10 times higher than that in the previous study and the virus vectors used in this study propagated faster than the previously used virus (rHEP5.0-CVSG-β-gal).

In both first- and second-order input areas, we mostly observed single-labeled neurons, even if the two populations showed some overlap. The relative sparseness of double-labeled cells is interpreted as supporting the topographical organization of both first- and second-order inputs. Although two viral vectors may fail to double-infect neurons because of competition, thus resulting in a false impression of topography [28], [29], it is unlikely that our results are biased as the result of viral competition. First, our single-virus tracing experiments, in which no competition occurred, showed similar topographical arrangements for both first- and second-order labeling. Second, our control experiments with more closely positioned injections in the dorsal DG showed that the two viral vectors are capable of producing dual infection of single neurons. In a previous study we reported that successful dual infection strongly depended on the timing of the occurrence of the two infections, which most importantly depended on the lengths of the axonal connections and the speed of axonal transport [29]. In case of the dorsal and ventral DG the lengths of the connectional pathways are likely to be similar and the speeds of axonal transport of the two vectors are similar. This thus would ensure dual infection of neurons projecting to both dentate targets.

The retrograde cell labeling that was noted in CA3, the entorhinal cortex, the medial septum, the diagonal band, and the supramammillary nucleus three days after injection in either the dorsal or ventral DG probably represents direct inputs to the DG. This conclusion is consistent with the results of tracing studies using more conventional (i.e., chemical rather than viral) anterograde or retrograde tracers [13]–[16], [21], [22], [33]–[35]. A few labeled neurons were also seen in the interpeduncular nucleus and the median raphe nucleus three days after infection. Previous tracing studies have shown that these areas have sparse direct projection to the DG [36]–[38]. However, since considerable numbers of labeled neurons are only seen five days after infection, we think that most of the labeling in the interpeduncular nucleus and the median raphe nucleus results from disynaptic labeling via the medial septum [36], [38]–[40]. The sparse labeling that we saw in the claustrum after three days probably represents second-order inputs already labeled after this shorter survival period. This conclusion is based on the fact that the labeling is very sparse and direct projections from this region to the DG have not been found in any of the conventional tracing studies. The second-order labeling in the claustrum is likely to be the result of dense innervation of the entorhinal cortex by this subcortical structure [41], [42]. The additional labeling that was seen in the piriform cortex and the endopiriform nucleus five days after virus injections likely represents second-order inputs because both of these structures have strong direct projections to the entorhinal cortex [43], [44]. The same is true for labeling seen after five days in the cingulate, prelimbic, and infralimbic cortices because projections from those regions to the DG have never been found in other tracing studies, while projections to the entorhinal cortex have been documented [45]–[47]. Although a weak direct projection from the presubiculum to the DG has been indicated by anterograde tracing [48], [49], we did not see this projection labeled three days after infection. Five-day survival, however, resulted in clear second-order labeling in the presubiculum, probably representing again inputs mediated by way of the entorhinal cortex; in this case, its medial subdivision [48], [50]. Unexpected is that we saw only sparse labeling in the perirhinal and postrhinal cortices as well as the amygdaloid complex, which all have strong projections to parts of the entorhinal cortex showing first-order labeling [45], [51]. This lack of labeling may be due to the fact that neurons may show selectivity in their uptake of viral tracers as is the case for DG neurons [29], [52]. We find this unlikely since we did see labeled neurons in these structures after longer survival times (i.e. 7 days). In addition, direct injection of the same vectors used in this study into the entorhinal cortex resulted in ample labeling of neurons in the perirhinal and postrhinal cortices after 3 days (unpublished results). Thus a more likely explanation is that these three inputs do not strongly innervate layer II principle cells. This is in accordance with the preferred laminar innervation of layer III of the entorhinal cortex by all inputs [45], [51]. However, this proposed explanation awaits further experimental evidence since projections from the presubiculum, also known to specifically distribute to layer III, reportedly also innervate layer II cells [53].

Other subcortical areas that according to our data send second-order projections to the DG are the lateral septum and the medial habenula, both structures that project to the medial septum [54]–[56]. When we injected viral vectors into the ventral DG, we saw many labeled neurons in the ventromedial and dorsal hypothalamic nucleus. This is likely to be due to inputs mediated either by way of the medial septum or the supramammillary nucleus [57], [58]. The tenia tecta, which was also labeled by the viral vectors injected into the ventral DG, is thought to have been indirectly labeled by way of the entorhinal cortex [44], [59].

### First-order topography maintained in second-order inputs

**Figure 12:**
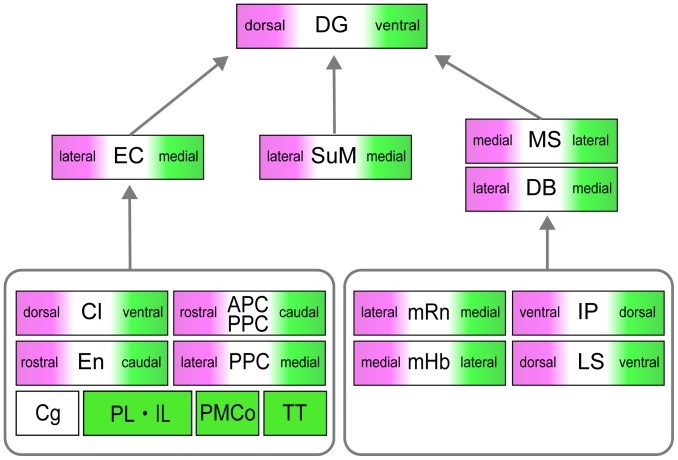
Summary of the mono- and disynaptic inputs to the dorsal and ventral hippocampus. Topographical projection patterns to the dorsal and ventral DG were seen in the first-order projection area and in most of the second-order projection area. Afferents of the dorsal DG are shown in magenta, and afferents of the ventral DG are shown in green. No topographical projection patterns were seen in the cingulate cortex (Cg), which has projection to both the dorsal and ventral DG. The prelimbic and infralimbic cortices (PL, IL), the posteromedial cortical area (PMCo), and the tenia tecta (TT) mainly project to the ventral DG. Abbreviations the same as in Figure 2 and 3.

Our most striking observation with respect to the second-order inputs to the DG is that they all show a clear topographical organization that seems to reflect that of the first-order projections (Fig. 12). The first-order labeling seen in the entorhinal cortex confirms a substantial literature indicating that the dorsoventral axis of the DG is mapped onto a dorsolateral-to-ventromedial axis of origin in the entorhinal cortex and holding true for both the lateral and medial entorhinal subdivisions [20]. This axis is reflected again in all inputs to the entorhinal cortex. In case of the piriform cortex, the axis runs from rostral to caudal, while for the posterior piriform cortex it maps from dorsolateral to ventromedial. In the claustrum, the dorsoventral DG axis is indirectly translated into a dorsoventral axis while for the endopiriform nucleus the translation is into a rostrocaudal one. In case of the posterior cortical nucleus of the amygdala our data are in line with reports that this area projects only to the rostromedial entorhinal cortex and not directly to the ventral DG [60]. In the medial prefrontal cortex we did not see a striking topographical organization. This contrasts with a recent study where such topography was found using pseudorabies trans-synaptic tracing [30]. In that study, cortical inputs to CA1 were described using a trans-synaptic tracing approach similar to the one we used in this study but the focus was on medial cortical areas such as the prelimbic, infralimbic, cingulate, and retrosplenial cortices. In view of the difference in injection areas, it is not surprising that several of the inputs described in the present paper were not reported in the CA1 study. This strongly indicates that viral tracing allows direct and indirect synaptic inputs to cortical areas to be studied selectively. The fact that our study did not replicate the reported topography in the medial prefrontal cortex points to slight differences between the organization of indirect medial prefrontal inputs to the DG and organization of indirect medial prefrontal inputs to CA1. This is likely to reflect a difference in the innervation of neurons in layers II and III, which are respectively the main sources of input to the DG and CA1. This may also explain the overall lower number of labeled cells in the medial cortical domains in our study, compared to the CA1 study, since inputs from the medial cortex preferentially distribute to layer V of the entorhinal cortex. Layer III cells extend basal dendrites into layer V and are therefore likely recipients of these inputs. In contrast, layer II cells only rarely have deep extending basal dendrites [61], [62].

In the medial septum, medially located neurons project to the dorsal DG and laterally positioned neurons project more ventrally. In the diagonal band this projection pattern is reversed and the neurons that project ventrally are ventromedial to those that project dorsally. The second-order inputs mediated through the medial septum-diagonal band complex include those originating in the lateral septum [55], [63], the medial habenula [54], [56], the median raphe nucleus [38], [56], and the interpeduncular nucleus [39], [40]. In the lateral septum the dorsoventral DG axis is related to a similarly oriented dorsoventral axis, in the medial habenula the orientation is from medial to lateral, in the median raphe nucleus the orientation is from lateral to medial, while in the interpeduncular nucleus the axis has a ventrodorsal orientation.

In the supramammillary nucleus, laterally located neurons project to the dorsal DG, while medially positioned neurons project more ventrally. This topographical projection patterns is in accordance with a previous study [35]. The major afferent inputs to the supramammilllary nucleus are from the infralimbic cortex, the dorsal peduncular cortex, the diagonal band, and the medial and lateral preoptic nuclei [64]. Thus, labeled neurons seen in the infralimbic cortex and the diagonal band five days after infection may include neurons infected disynaptically by way of the supramammillary nucleus.

### Functional implications

The connectional differences along the long axis of the dentate gyrus are complemented by connectional gradients in the connectivity of the other hippocampal subfields as well as differences in intrinsic connections [20]. Differences in the percentages of principal neurons and interneurons [65], [66] and distributions of corticosteroid receptors along the long hippocampal axis have been reported [67], as well as differences in gene expression patterns [68]–[70]. The firing fields of place cells in the hippocampus increase their dimensions from dorsal to ventral [71], and the spacing of firing peaks of single grid cells in the medial entorhinal cortex increases along a dorsoventral axis [72], [73]. There is ample experimental evidence in support of functional differences along the long axis of the hippocampus [74], [75]. This is most strikingly shown by lesion studies in the rat, where functional deficits caused by dorsal lesions differ from those resulting from ventral lesions: dorsal lesions resulting in spatial deficits and ventral lesions instead resulting in changes in fear-related responses [10], [76], [77]. A comparable behavioral difference has been reported by investigators comparing the results of lesions in the caudolateral entorhinal cortex with those of lesions in the rostromedial domains of the entorhinal cortex [78]. These functional differences have often been thought to reflect the differences in intrinsic and extrinsic wiring summarized above. The present results clearly show that these functional differences go beyond these primary features and include multisynaptic input pathways.

For some of those multisynaptic routes, differences have been reported along the axes observed in the present study. These functional differences seem to focus around two main themes, and for both the topographies observed in first- and second-order input regions are in accordance with each other. First, the dorsal and ventral hippocampus differ with respect to the firing characteristics of neural populations in that theta power decreases from dorsal to ventral [79]. Since the medial septum, diagonal band of Broca, and the supramammillary nucleus play important roles in the generation of theta rhythm, the observed topological relationships [54], [80] may be related to the differences in theta power. In the medial septum, noncholinergic neurons are mainly located in the medial half, whereas cholinergic neurons are mainly in the lateral half [81]. Thus the dorsal hippocampus receives inputs from the noncholinergic medial septal neurons and cholinergic diagonal band neurons, whereas the ventral hippocampus receives inputs from the cholinergic medial septal neurons [82], [83]. Likewise, the projection from the lateral region of the supramammillary nucleus to dorsal DG originates from both GABAergic and glutamatergic neurons, whereas the projection from the medial part of the supramammillary nucleus to ventral DG displays a glutamatergic phenotype [84]. Serotonergic projections from the median raphe nucleus have been implicated in synchronizing hippocampal and septal activity [38], but nothing is known about a functional difference between medial and lateral raphe parts.

Second, dorsal and ventral parts of the hippocampus differ with respect to the overall quality of the information they process. The dorsal hippocampus apparently interacts with brain structures that are part of the realm that processes exteroceptive information. The ventral hippocampus, in contrast, through its first order connected structures, is strongly implicated in motivational and reward systems of the brain. The present data indicate that this focus is also reflected in the second-order inputs. The medial part of posterior piriform cortex receives strong input from the amygdaloid complex [85], similar to the preferred connectivity of the ventral hippocampus, thus strongly suggesting that the stream from the medial part of the posterior piriform cortex to the ventral DG carries olfactory information related to emotional events. The preferred projection of the posterior cortical nucleus of the amygdala to the ventral hippocampus fits into this pattern as well. Along similar lines, the preferred localization of tyrosine hydroxylase-positive neurons in the medial part of the supramammillary nucleus suggests that the ventral hippocampus probably receives stronger dopaminergic inputs than the dorsal hippocampus [86], reflecting a preferred relation of the ventral hippocampus with the reward system.

The topographically distributed labeling in the lateral septum mirrors the dorsoventral distributions of hippocampal projections to the lateral septum. This organization has been suggested to reflect a functional relationship with functionally different hypothalamic domains, such that the ventral hippocampus is connected with hypothalamic nuclei involved in the modulation of endocrine and autonomic responses, whereas the dorsal hippocampus is connected with hypothalamic domains relevant for exploratory behavior and behavioral arousal [55]. To our knowledge, the functional significance of the other second-order input areas, such as the medial habenula, interpeduncular nucleus, endopiriform nucleus, and the claustrum, have hardly been studied.

In conclusion, we show that the topographical organization of the two different connectional chains into the dorsal and ventral DG is consistent for both first-order and second-order inputs and fits with reported and hypothesized functional differences along the hippocampal dorsoventral axis. Recent findings that the dorsoventral differentiation in the hippocampus is established early during development [87] suggests that the topographically organized connectional chains reported here are established during early development of the brain. Although a mechanistic understanding is still lacking, these different connectional chains that go beyond direct input relations may form an important factor contributing to adult functional differences along the hippocampal dorsoventral axes.
